# Trends in Outcomes for Out-of-Hospital Cardiac Arrest by Age in Japan: An Observational Study

**DOI:** 10.1097/MD.0000000000002049

**Published:** 2015-12-11

**Authors:** Tatsuma Fukuda, Naoko Ohashi-Fukuda, Takehiro Matsubara, Kent Doi, Yoichi Kitsuta, Susumu Nakajima, Naoki Yahagi

**Affiliations:** From the Department of Emergency and Critical Care Medicine, Graduate School of Medicine, The University of Tokyo, Hongo, Bunkyo-ku, Tokyo, Japan.

## Abstract

Population aging has rapidly advanced throughout the world and the elderly accounting for out-of-hospital cardiac arrest (OHCA) has increased yearly.

We identified all adults who experienced an out-of-hospital cardiac arrest in the All-Japan Utstein Registry of the Fire and Disaster Management Agency, a prospective, population-based clinical registry, between 2005 and 2010. Using multivariable regression, we examined temporal trends in outcomes for OHCA patients by age, as well as the influence of advanced age on outcomes. The primary outcome was a favorable neurological outcome at 1 month after OHCA.

Among 605,505 patients, 454,755 (75.1%) were the elderly (≥65 years), and 154,785 (25.6%) were the oldest old (≥85 years). Although neurological outcomes were worse as the age group was older (*P* < 0.0001 for trend), there was a significant trend toward improved neurological outcomes during the study period by any age group (*P* < 0.005 for trend). After adjustment for temporal trends in various confounding variables, neurological outcomes improved yearly in all age groups (18–64 years: adjusted OR per year 1.15 [95% CI 1.13–1.18]; 65–84 years: adjusted OR per year 1.12 [95% CI 1.10–1.15]; and ≥85 years: adjusted OR per year 1.08 [95% CI 1.04–1.13]). Similar trends were found in the secondary outcomes.

Although neurological outcomes from OHCA ware worse as the age group was older, the rates of favorable neurological outcomes have substantially improved since 2005 even in the elderly, including the oldest old. Careful consideration may be necessary in limiting treatment on OHCA solely for the reason of advanced age.

## INTRODUCTION

Population aging has rapidly advanced throughout the world.^1–3^ Whether treatment of elderly patients should be restricted has become an important issue, not only for chronic diseases but also emergency and critical care.^4,5^ Treatment for out-of-hospital cardiac arrest (OHCA) is no exception.^6,7^

OHCA is one of the worst diseases. It occurs in approximately 120,000, 280,000, and 330,000 people a year in Japan, the EU, and the United States, respectively.^8–12^ The percentage of elderly patients accounting for OHCA has increased yearly.^13–15^ In Japan, where population aging has progressed most in the world,^1–3^ it is urgent to address how we should manage elderly OHCA patients. However, information is still too limited to examine whether the treatment for elderly OHCA patients should be restricted.

At present, in Japan, once the emergency medical service (EMS) system is called into action for OHCA, resuscitation efforts are equally conducted on almost all OHCA patients, regardless of age, at least until they are transferred to an emergency and critical care center. All data for these OHCA patients are recorded and maintained by the Fire and Disaster Management Agency (FDMA) of the Ministry of Internal Affairs and Communications.

This study aimed to investigate temporal trends in rates of favorable outcomes for OHCA patients by age and to examine the influence of advanced age on outcomes, using population-based OHCA data of FDMA from 2005 to 2010 in Japan.

## METHODS

### Data Source

The All-Japan Utstein Registry of the FDMA is a nationwide, prospective, population-based clinical registry of patients with OHCA in Japan. The design of the registry has been described in detail previously.^8^ Briefly, all patients with a confirmed OHCA (defined as the absence of a palpable central pulse, apnea, and unresponsiveness) of all causes and for whom resuscitation is attempted are identified and followed, including those with do-not-resuscitate (DNR) orders. Data are collected from 3 sources that together define the continuum of emergency cardiac care: 119 dispatch centers, EMS agencies, and receiving hospitals. The registry uses standardized Utstein-style definitions for clinical variables and outcomes to ensure uniformity.^16,17^ Data completeness and accuracy is ensured by rigorous certification of hospital staff and use of standardized software with internal data checks.

This study was conducted in accordance with the amended Declaration of Helsinki. The FDMA and the Institutional Review Board at The University of Tokyo approved this study with a waiver of informed consent because of the anonymous nature of the data.

### Study Population

**FIGURE 1:**
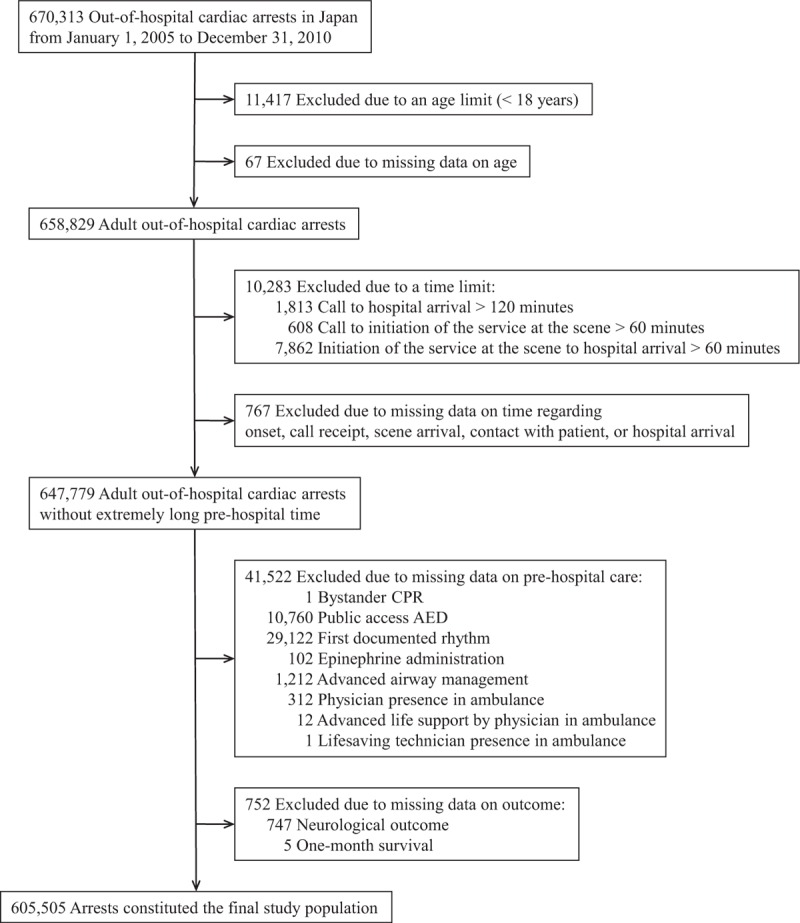
Study cohort. AED = automated external defibrillator; CPR = cardiopulmonary resuscitation.

Our analysis was based on 670,313 cases submitted to the All-Japan Utstein Registry of the FDMA from January 1, 2005, through December 31, 2010 (Fig. 1). We excluded 11,417 patients who were younger than 18 years of age, and we also excluded 67 patients with missing data on age. We restricted our sample to patients without an extremely long prehospital time (time from call to initiation of the service at the scene ≤60 minutes, time from initiation of the service at the scene to hospital arrival ≤60 minutes, and time from call to hospital arrival ≤120 minutes), and excluded 10,283 patients because patients who require an extremely long prehospital time have distinct prehospital circumstances and outcomes. We also excluded 767 patients with missing data on time regarding onset, call receipt, scene arrival, contact with patient, or hospital arrival. Furthermore, we also excluded patients with missing data on prehospital care: bystander cardiopulmonary resuscitation (CPR) (1 patient), public access automated external defibrillator (AED) (10,760 patients), first documented rhythm (29,122 patients), epinephrine administration (102 patients), advanced airway management (1,212 patients), physician presence in ambulance (312 patients), advanced life support by physician in ambulance (12 patients), or emergency lifesaving technician presence in ambulance (1 patient). Finally, we excluded patients with missing data on outcomes: neurological outcomes (747 patients) or 1-month survival (5 patients). Our final sample comprised 605,505 patients.

### Study Outcomes

The primary outcome was a favorable neurological outcome at 1 month after OHCA, measured by the Glasgow–Pittsburgh cerebral performance category (CPC) scores. A CPC score of 1 or 2 (good performance or moderate disability, respectively) was defined as a favorable neurological outcome, and a CPC of 3, 4, or 5 (severe disability, vegetative state, or death, respectively) was regarded as an unfavorable neurological outcome.^17,18^ We analyzed temporal trends in the rate of favorable neurological outcome in the overall cohort and separately according to the 3 age groups: 18 to 64, 65 to 84, and ≥85 years. As secondary outcomes, we analyzed temporal trends in the rate of prehospital return of spontaneous circulation (ROSC) and those of 1-month survival. To assess whether any recent temporal trends in favorable neurological outcome could only depend on those with 1-month survival or could be attributed to advances in in-hospital or postresuscitation care, we also examined rates of favorable neurological outcome among 1-month survivors.

### Statistical Analysis

The baseline characteristics of the study cohort were described with the use of proportions for categorical variables and means with standard deviations for continuous variables. To evaluate changes in baseline characteristics by calendar year, we used the Cochran–Armitage trend test for categorical variables and linear regression for continuous variables.

To assess whether outcomes had improved over time, multivariable logistic regression analysis was used. Our independent variable, calendar year, was included in the model as a categorical variable, with 2005 to 2006 as the reference period. We also evaluated calendar year as a continuous variable to obtain adjusted odds ratios for year-to-year trends in the rate of favorable outcomes. We also examined whether trends in the rate of favorable outcomes differed by age group.

In addition to calendar year (2005–2006, 2007–2008, or 2009–2010) and age (18–64, 65–84, and ≥85 years), our models adjusted for sex (male or female), witness (presence or absence), bystander CPR (presence or absence), public access AED (presence or absence), first documented rhythm (shockable rhythm or nonshockable rhythm), etiology of arrest (cardiac etiology or noncardiac etiology), advanced airway management (presence or absence), epinephrine administration (presence or absence), physician in ambulance (presence or absence), time from call to contact with patient, and time from call to hospital arrival.

Stratified subgroup analyses were performed to assess the influence on trend in neurologically favorable survival rates according to witness, first documented rhythm, and etiology of arrest.

All statistical analyses were conducted using JMP Pro 11.0.0 software (SAS Institute Inc, Cary, NC). All hypothesis tests were 2-sided with a significant level of 0.05.

## RESULTS

Among 605,505 patients, 454,755 (75.1%) were the elderly (≥65 years). In particular, 154,785 (25.6%) were the oldest old (≥85 years). During the study period, the proportion of the elderly OHCA increased from 73.0% in 2005 to 77.3% in 2010 (*P* < 0.0001 for trend), and the proportion of the oldest old OHCA increased 23.1% in 2005 to 28.4 in 2010 (*P* < 0.0001 for trend). Temporal trends in baseline characteristics of the study cohort, grouped into 3 time periods, are summarized in Table 1. The mean age of the study population was 73.1 years (standard deviation of 16.2 years), and 58.4% were men. There were modest differences over time in all baseline characteristics as well as the age (*P* < 0.05 for trend for all comparisons).

**TABLE 1:**
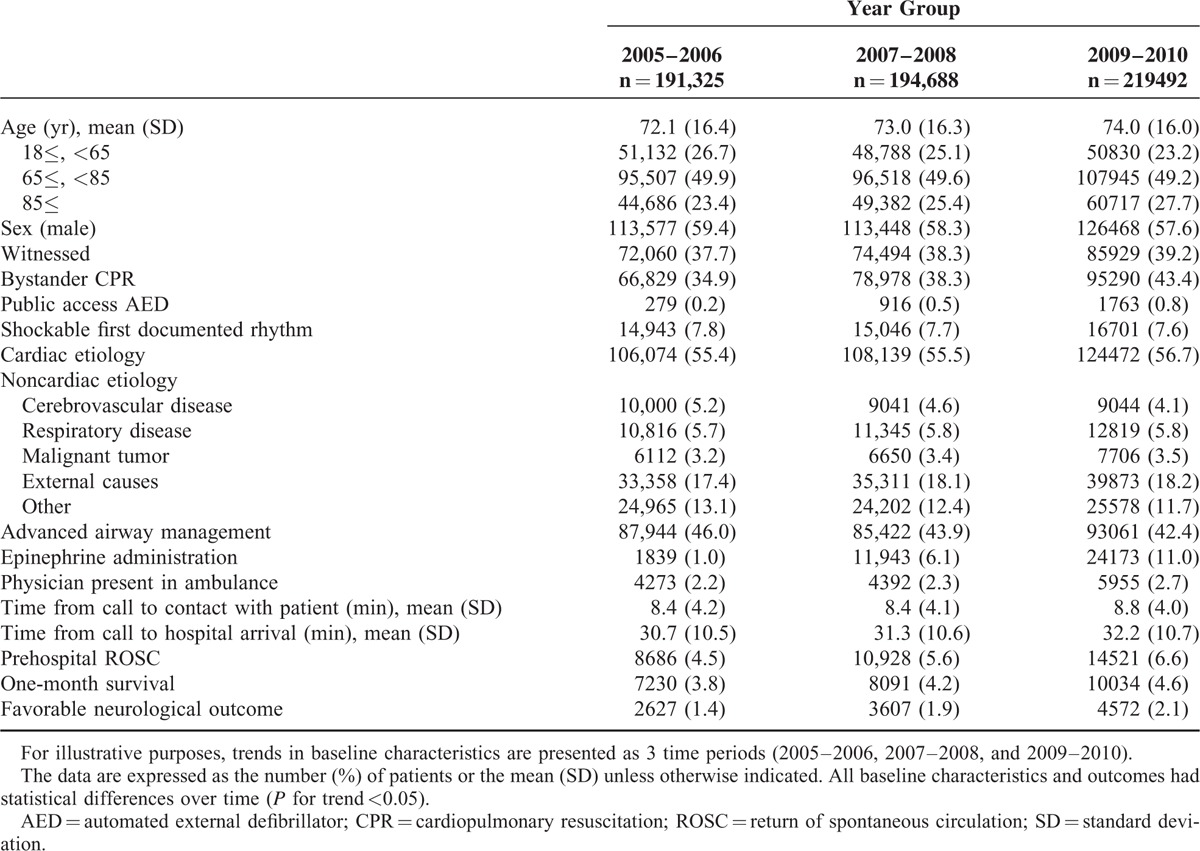
Trends in Baseline Characteristics in Patients With an Out-of-Hospital Cardiac Arrest

### Favorable Neurological Outcome

The overall rate of favorable neurological outcome was 1.8% (10,806 of 605,505 patients). There was a significant trend toward increased favorable neurological outcome during the study period for all study patients, as well as for any of the 3 age groups (Tables 2 and 3 ). After adjustment for temporal trends in patient, cardiac arrest, procedural, and prehospital characteristics, overall favorable neurological outcome increased from 1.4% in 2005 to 2.1% in 2010 (adjusted odds ratio per year, 1.12; 95% confidence interval, 1.11–1.14). Neurological outcomes ware worse as the age group was older through the study period (*P* < 0.0001 for trend). However, depending on the age group, neurological outcome improved yearly even in the elderly, including the oldest old (65–84 years: adjusted odds ratio per year 1.12, 95% confidence interval 1.10–1.15; and ≥85 years: adjusted odds ratio per year 1.08, 95% confidence interval 1.04–1.13).

**TABLE 2:**
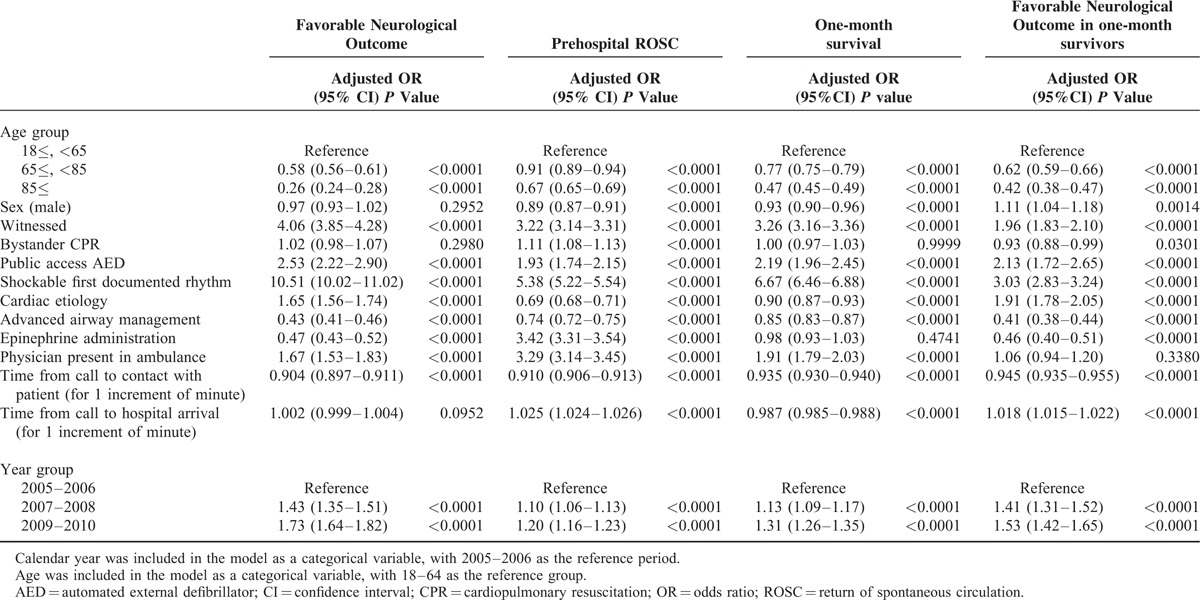
Contributing Factors to Favorable Outcomes in Patients With an Out-of-Hospital Cardiac Arrest

**TABLE 3:**
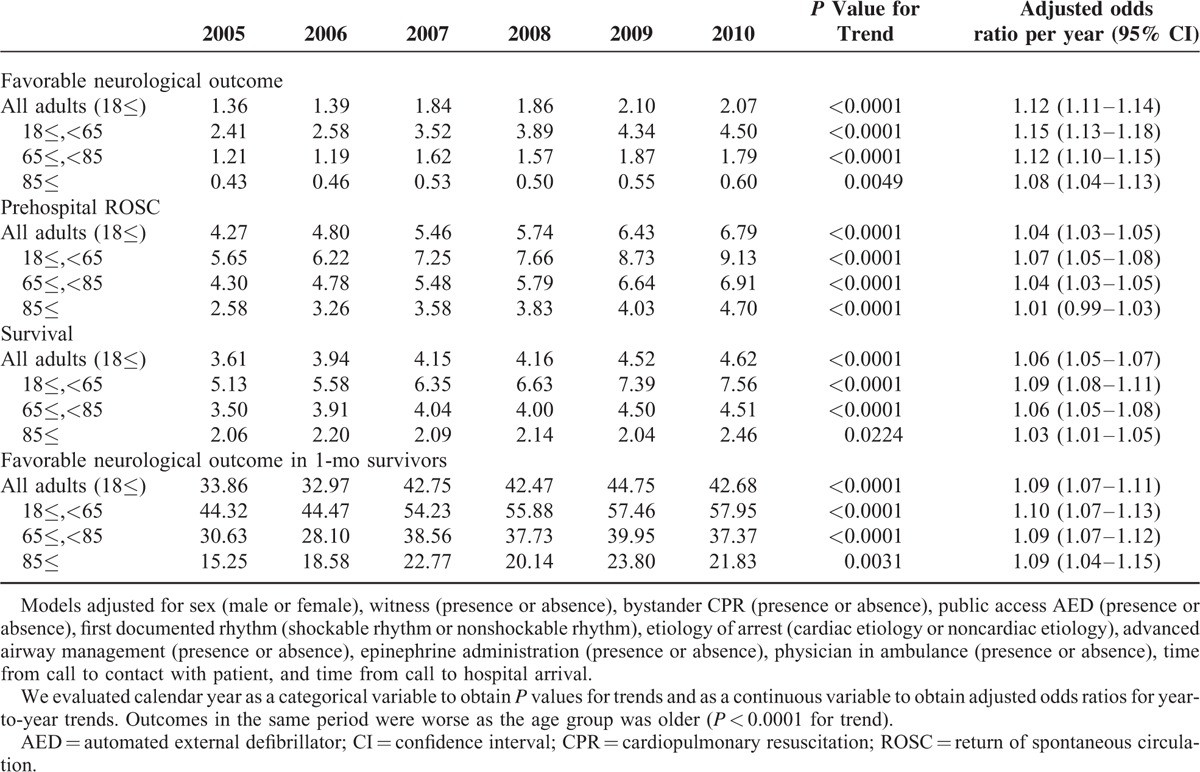
Trends in Rates of Favorable Neurological Outcomes, Prehospital ROSC, 1-Mo Survival, and Favorable Neurological Outcomes Among 1-Mo Survivors

### Secondary Outcomes

We examined temporal trends in the rate of prehospital ROSC and those of 1-month survival (Tables 2 and 3). Both prehospital ROSC and 1-month survival showed similar trends, as did neurological outcome.

We also examined the rates of favorable neurological outcome among 1-month survivors, to evaluate whether the increased rates of favorable neurological outcome just reflected the increased survival rate or implied that in-hospital or postresuscitation care had advanced over the study period. The results showed an improving yearly trend, not only for all patients but also for each age group.

In addition, we conducted stratified subgroup analyses to assess whether the trends in neurologically favorable survival rates of OHCA differ depending on witness, first documented rhythm, and etiology of arrest (Table 4). Yearly improvement in neurologically favorable survival rates was greater when a witness, shockable initial rhythm, or cardiac etiology existed in any subgroups other than the oldest old. In the oldest old subgroup, the first documented rhythm and the etiology of arrest had little influence on improving trends in neurologically favorable survival rates.

**TABLE 4:**
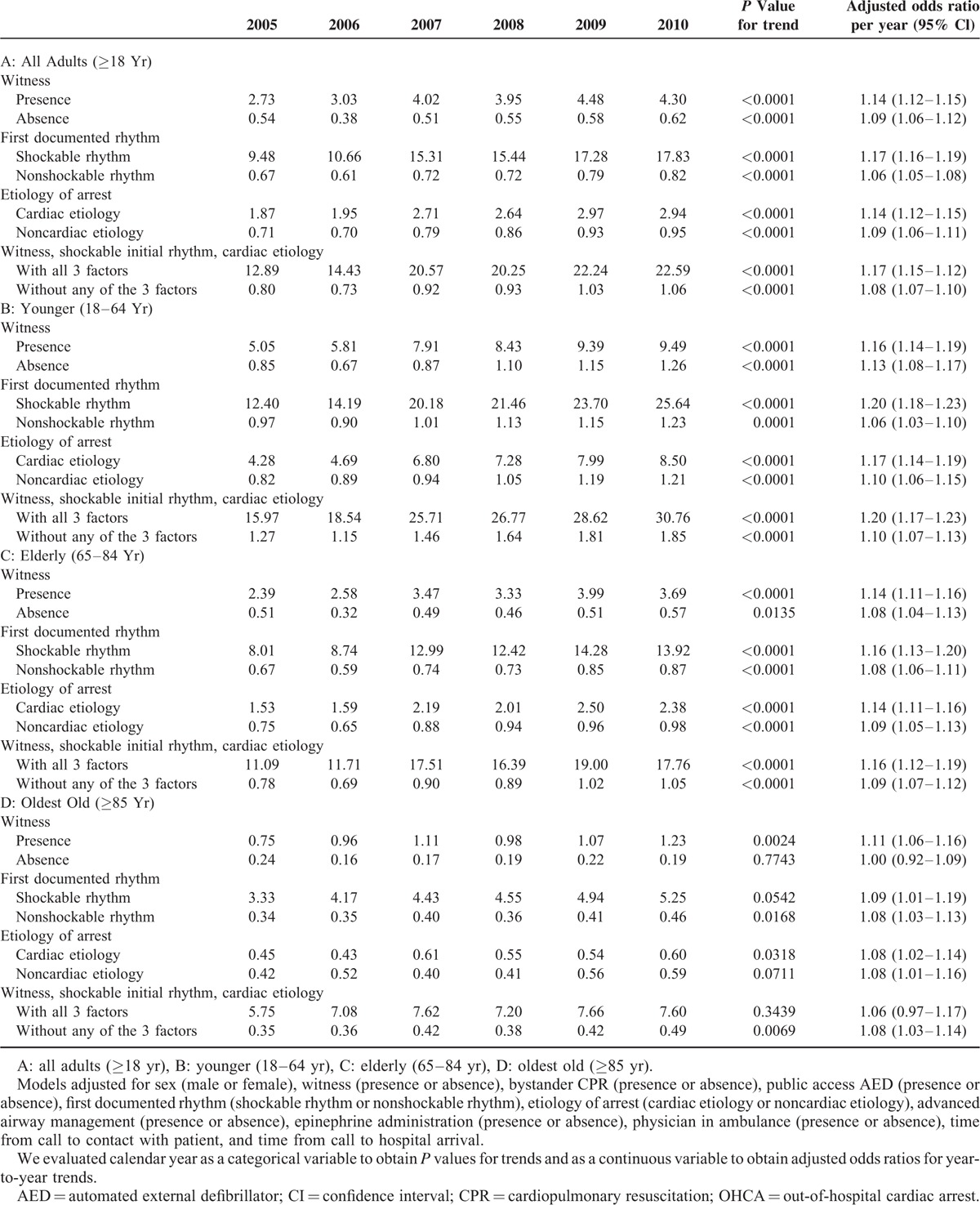
Trends in Rates of Favorable Neurological Outcomes for OHCA Stratified by Witness, First Documented Rhythm, and Etiology of Arrest

## DISCUSSION

Based on data collected from a nationwide, prospectively collected population-based registry of patients with OHCA in Japan, we found that overall rate of favorable neurological outcome after OHCA improved substantially between 2005 and 2010. There were accompanying improved trends in the oldest old as well as in the elderly, although neurological outcome from OHCA worsened as the age groups grew older.

Several studies have reported improved rates of favorable outcomes in cardiac arrest patients. However, most of them have not necessarily focused on elderly OHCA.^13–16,19–23^ Even when elderly cardiac arrest patients were dealt with, most studies have only handled in-hospital cardiac arrest^24–26^ or OHCA in a limited population.^27–29^ Therefore, our findings regarding trends of outcomes in elderly OHCA patients derived from a nationwide population-based registry data are highly valuable.

In Japan, except in specific situations (eg, decapitation, rigor mortis, livor mortis, or decomposition), resuscitation efforts are conducted on all OHCA patients equally, at least in the prehospital setting, and all data for these OHCA patients are recorded and maintained by the FDMA. Thus, we were able to investigate temporal trends of outcomes of OHCA patients by age and to examine the influence of advanced age on outcomes using those registry data.

Our findings will provide information useful not only for Japan but also any other countries confronted with population ageing.

In our study, neurological outcomes showed improving trends even in the oldest old as well as in the elderly. However, the outcome in the same period was worse as the age group was older, and the improvement of outcome over time was less as the age group was older. The reason that the outcome in the same period was worse as the age group was older might be related to poor physiological function or comorbidities in the elderly.^30^ Alternatively, the elderly might tend to have restrictions on treatment or DNR orders.^6,31^ Our findings that the improvement in outcome over time was reduced with older age groups might imply that there was little room for improvement despite advances in OHCA treatment; even if there was room for improvement, as in the younger age group, over time improvement in older age groups might be reduced because of the possible yearly increase in restrictions on treatment or DNR orders. In our study, details on this information were not available. For more rigorous examination, further study including such information will be necessary.

In our study, not only neurological outcome but also prehospital ROSC showed an improving trend for each age group. However, although the unadjusted rate of prehospital ROSC showed a significant improving trend, no significant improving trend of prehospital ROSC rate was found after adjustment for each quantitative confounding factor in the oldest old. In the oldest old group, improvement of outcome by qualitative alterations in prehospital care (eg, bystander CPR or public-access defibrillation) may approach the limit. However, considering that the implementation rates of bystander CPR or public-access defibrillation are still low, improvement of outcome by quantitative alteration may be expected.

In our study, 1-month survival showed an improving trend for each age group, as did neurological outcome. Therefore, we also examined the percentage of favorable neurological outcome among 1-month survivors to evaluate whether the improving trend of neurological outcome reflected just the increase in survival rate or implied that in-hospital or postresuscitation care has advanced. The results showed a yearly improving trend both for all patients and also for each age group. This suggested that advanced in-hospital or postresuscitation care might contribute to improvement of outcomes even in the elderly including the oldest old. However, the increased percentage of favorable neurological outcome among 1-month survivors may not always be due to advanced in-hospital or postresuscitation care. For instance, if restrictions on treatment or DNR orders for people in whom a poor neurological outcome is anticipated increased through the study period, and the survival rate increased nonetheless, the percentage of favorable neurological outcome among 1-month survivors would increase even if in-hospital or postresuscitation care has not advanced. For more accurate evaluation, further studies including detailed information on in-hospital or postresuscitation care and information about restrictions on treatment or DNR orders will be necessary.

Based on the recent trend, neurological outcome in OHCA has continually improved even in the oldest old, although it was worse with older age groups. In addition, as shown in our subgroup analyses, some factors other than age could affect improving trends in neurologically favorable survival rates. Therefore, we should not readily set a limit on treatment solely for the reason of advanced age.

It may also be significant that prehospital care is linked to in-hospital or postresuscitation care without termination of resuscitation in the prehospital setting because the improvement in trends of neurologically favorable survival rates may have been caused critically from the advance in in-hospital or postresuscitation care.

However, no matter how the improving trend was shown, the outcome for the oldest old in all OHCA was remarkably poor compared with younger age groups. Therefore, to judge whether treatment for elderly OHCA, especially OHCA in the oldest old, is beneficial or futile, examination with subdivision of the groups by adding other factors, besides age, or cost-effectiveness analysis will also be required.

Our study is limited in certain respects. First, the All-Japan Utstein Registry of the FDMA collects merely data of essential items. Hence, detailed clinical information to evaluate other factors (such as comorbidities) that might influence outcomes is unavailable. Although aging of OHCA has advanced through the study period, the health of the elderly that may influence outcomes may have also improved thanks to the advance of medicine. Second, the data from the All-Japan Utstein Registry do not cover everything of resuscitation care. It includes information on only prehospital care, and not on in-hospital or postresuscitation care. The information of prehospital care is only quantitative data, not qualitative data. In addition, for in-hospital or postresuscitation care, although there are factors that may strongly influence the outcome (eg, targeted temperature management, cardiac catheterization, implementation of extracorporeal life support, or DNR orders), these data are not available. The possibility of residual confounding still remains. Third, although neurological outcomes were evaluated by the use of a CPC score in each facility, judgment what category a particular neurological outcome corresponded to may be somewhat subjective. Fourth, the findings of this study indicate the association, but not necessarily the causality. In Japan, almost all OHCA patients receive resuscitative care. Although the All-Japan Utstein Registry data included all types of OHCA, there were assumptions that the same resuscitative efforts were attempted to all registered patients in conduct of this study. However, same resuscitative efforts might not be provided for all OHCA patients. If EMS personnel or clinicians were aware of differences in outcomes in the elderly (≥65 years) or patients without prehospital ROSC, this awareness might affect the quality of prehospital, in-hospital, or postresuscitation care. Finally, like other epidemiological studies, there are potential limitations in data integrity, validity, and ascertainment bias. To minimize these potential sources of bias, we use a uniform data record consistent with Utstein-style guidelines for reporting cardiac arrest, the large sample size, and the population-based design.

In conclusion, in a nationwide, prospective, quality improvement registry, we found that rates of favorable neurological outcome from OHCA in Japan have substantially improved since 2005 in not only overall adults but also the elderly and the oldest old OHCA, although neurological outcome from OHCA was worse as the age group was older. This improvement was accompanied by increased rates of favorable neurological outcome among 1-month survivors over time. Careful consideration may be necessary in limiting treatment on OHCA solely for the reason of advanced age.
